# Phenotyping multiple subsets in Sjögren’s syndrome: a salivary proteomic SWATH-MS approach towards precision medicine

**DOI:** 10.1186/s12014-019-9245-1

**Published:** 2019-06-20

**Authors:** Antonella Cecchettini, Francesco Finamore, Nadia Ucciferri, Valentina Donati, Letizia Mattii, Enza Polizzi, Francesco Ferro, Francesca Sernissi, Marta Mosca, Stefano Bombardieri, Silvia Rocchiccioli, Chiara Baldini

**Affiliations:** 1 National Research Council – Clinical Institute of Physiology, Pisa, Italy; 2 Unit of Anatomic Pathology II, Azienda Ospedaliero-Universitaria Pisana, Pisa, Italy; 3 Section Histology, Department of Clinical and Experimental Medicine, University of Pisa, Pisa, Italy; 4 Rheumatology Unit, Department of Clinical and Experimental Medicine, University of Pisa, Pisa, Italy

**Keywords:** Sjögren’s syndrome, Salivary proteomics, Mass spectrometry, SWATH-MS, Biomarkers, Precision medicine

## Abstract

**Background:**

This proof of concept study was aimed at characterizing novel salivary biomarkers specific for different subsets in primary Sjögren’s syndrome (pSS) in order to improve patients’ profiling.

**Methods:**

pSS patients were stratified in three subgroups according to both (a) focus score in the minor salivary gland biopsies (i.e. intensity of immune cell infiltration in the tissue) and (b) unstimulated salivary flow rate. Healthy volunteers were included as controls. A nano-HPLC-SWATH-MS approach was used for the analysis of saliva proteome of different subsets.

**Results:**

We found 203 differentially expressed proteins in pSS patients with respect to controls with evident differences in the expression of normal constituents of the human salivary proteome (i.e. prolactin-inducible protein, proline-rich proteins, cystatins) and several mediators of inflammatory processes. The comparative analysis of the pSS phenotypes unrevealed 63 proteins that were shared and specifically modulated in the three subsets of pSS patients converging on several inflammatory pathways. Among them S100A protein appeared of particular interest merging on IL-12 signaling and being significantly influenced by either salivary flow impairment or intensity of immune cell infiltration in the tissue.

**Conclusions:**

Constellations of proteins, including S100A proteins, characterize different pSS subsets reflecting either salivary gland dysfunction or inflammation. Salivary proteomics may foster future research projects ultimately aimed at developing personalized treatments for pSS patients.

**Electronic supplementary material:**

The online version of this article (10.1186/s12014-019-9245-1) contains supplementary material, which is available to authorized users.

## Background

Primary Sjögren’s syndrome (pSS) is a complex autoimmune disease characterized by a wide spectrum of clinical features, ranging from inflammation and hypo-function of salivary and lachrymal glands to severe multi-systemic organ involvement, potentially evolving into malignant lymphomas [1–5]. In fact, a significant inter-subject variability has been described in pSS also at glandular level, with patients presenting a great deal of variation in the intensity of their minor salivary gland biopsy (MSGB) infiltrates and in salivary flow production [6–8].

Recently, a growing interest has arisen in pSS patient stratification in order to move towards personalized treatments. To identify reliable biomarkers able to distinguish pSS sub-groups, salivary “omics” techniques have been in the spotlight as novel, valuable tools [9–12]. Several candidate biomarkers have been proposed including numerous defense proteins, like salivary immunoglobulins, cationic peptides, lysozyme, prolin-rich proteins, mucins and cystatins [12–25]. Despite these encouraging results, however, translation into clinical practice of the identified putative biomarkers has not been accomplished and particularly, pSS inter-subject variability and heterogeneity have not been addressed [26].

In this proof of concept study a high-throughput liquid chromatography coupled to a data-independent sequential window acquisition of all theoretical fragment ion spectra (SWATH-MS) approach was used to search for salivary proteomic biomarkers in pSS specific subsets. For the purpose of this study patients were stratified on the basis of (1) the complexity of lymphocytic infiltration detected in MSGB and (2) on the variation of their unstimulated salivary flow rate (USFR).

The ultimate aim of this study was to contribute to the comprehension of the complexity of the disease, through the identification of novel salivary biomarkers potentially related to pSS different subsets characterized by distinctive pathophysiological processes.

## Materials and methods

### Patient recruitment

To identify proteomic patterns specific to salivary gland inflammation or dysfunction, we included in this study patients with pSS (AECG 2002) [27] who had low-grade or high-grade inflammation in their salivary gland biopsies and impaired or normal saliva production. More specifically, according to the literature [28, 29], we defined the level of tissue inflammation on the basis of the focus score in the gland biopsies, considering a focus score ≥ 3 as high, and the unstimulated salivary flow as decreased when lower than 1.5 ml/15 min. Patients were therefore, subgrouped in three different disease phenotypes characterized by high-grade inflammation and normal unstimulated salivary flow rate (i.e. high focus/normal flow), high-grade inflammation and decreased unstimulated salivary flow rate (i.e. high focus/low flow) or low-grade inflammation and decreased unstimulated salivary flow rate (i.e. low focus/low flow), respectively.

Exclusion criteria were the presence of signs or symptoms of periodontitis and a USFR < 0.2 ml/min [23]. Moreover, we decided not to include pSS patients with mild infiltrates and USFR ≥ 2.5 in order to restrict the analysis to subjects with at least either histopathological or functional findings suggestive for the diagnosis of pSS. A total of twenty patients were included in the study. And age- and sex-matched healthy volunteers were used as controls.

### Salivary samples collection and pre-processing

Salivary samples were collected according to a standardized protocol [15, 18]. Briefly, unstimulated whole saliva was collected between 9 and 11 a.m. from patients who had refrained from eating or drinking for 2 h. Immediately after collection and salivary flow rates determination, samples were centrifuged at 2000×g for 20 min at 4 °C to remove debris and cells, and protein concentration determined by Protein Assay dye reagent (Bio-Rad; Richmond, CA). Samples belonging to the same group were pooled in duplicate and stored at − 80 °C until analysis.

### Minor salivary gland samples collection and histopathologic analysis

MSGBs were obtained as part of routine diagnostic procedures in pSS patients, fixed in neutral buffered formalin, paraffin-embedded and H&E stained. All the samples were re-evaluated by an expert pathologist for FS assessment.

### Proteomics sample processing

Albumin and IgG were removed from saliva specimens by immunoaffinity chromatography using the ProteoPrep Immunoaffinity Albumin and IgG depletion kit (Sigma Aldrich, St. Louis, USA). 20 µg of depleted proteins were dissolved in Ammonium Bicarbonate 25 mM, reduced using dithiothreitol 5 mM at 80 °C for 20 min and alkylated with iodoacetamide 10 mM in dark at 37 °C for 30 min. Digestion was obtained incubating overnight with 1:100 trypsin (Roche, Germany): substrate at 37 °C. Peptide solutions were acidified and then loaded on a C18 cartridge in order to eliminate debris and additionally cleaned with 0.22 μm filters. Peptides were diluted to 0.1 µg/µL by 2% Acetonitrile (ACN, Romil, UK)/0.1% Formic Acid (FA); 5 µL were injected for library searching and 2 µL in duplicate for SWATH™ method analysis.

### NanoLC–MS/MS SWATH analysis

Chromatographic separation of peptides was performed using a nano-HPLC system (Eksigent, ABSciex, USA) with a loading pump that pre-concentrated the sample in a pre-column cartridge (PepMap-100 C18 5 µm 100 A, 0.1 × 20 mm, Thermo Scientific, USA). Then separation was done in a C18 PepMap-100 column (3 µm, 75 µm × 250 mm, Thermo Scientific, USA) at a flow rate of 300 nL min^−1^. Runs were performed with eluent A (Ultrapure water, 0.1% FA) under 60 min linear gradient from 5 to 40% eluent B (ACN/0.1% FA) followed by 10 min of a purge step and 20 min re-equilibration step.

Peptides eluted from chromatography were directly processed using TripleTOF™ 5600+ mass spectrometer (ABSciex, USA) equipped with a DuoSpray™ ion source. Data were acquired using a SWATH-MS method for large scale data independent MRM acquisition. An Information Dependent Acquisition (IDA) method was first generated and used to acquire the samples, in order to build a comprehensive spectral ion library for subsequent MRM processing. SWATH-MS acquisitions were performed over a mass range of 400–1250 m/z split into 35 overlapping isolation mass windows of 26 Da each (25 Da/mass selection for optimal ion transmission efficiency and 1 Da of window overlap). Peptide activation was performed using CID, using nitrogen as inert gas, with rolling collision energy and 5 eV of energy spread. The accumulation time was set to 0.25 s for MS1 and 90 ms for MS2 scan. The entire duty cycle was approximately 3.2 s. Maximum resolving power was reached at 20,000 enabling the extraction of fragment ion within an accuracy of 10–30 ppm.

For library, MS/MS data were processed with ProteinPilot™ Software (ABSciex, USA). The false discovery rate (FDR) analysis was set to a confidence level of 95%.

The label free statistical comparative analysis was performed using PeakView™ Software with MS/MS (ALL) with SWATH™ Acquisition MicroApp 2.0 and MarkerView™(ABSciex, USA). Retention time alignment was obtained using selected peptides (top confidence and top level transitions) from top score protein. Processing settings were: 7 peptides per protein, 7 transitions per peptide, 92% peptide confidence (according to Paragon algorithm result) and 1% FDR; XIC options: extraction window 10 min, width 50 ppm and 0.1 Da.

Fragment ion abundances were extracted for each matched peptide and integrated together in order to obtain the peptide abundance. Proteins abundances were calculated by summing the abundances of their specific peptides and normalization was performed using the total ion current (TIC) extracted from the full MS1 survey scan acquisition for each run.

### Gene ontology (GO) terms analysis

All differentially expressed proteins commonly found in the three pSS phenotypes (group A, B and C) were categorized for GO annotations using the ClueGo plug-in [30] of Cytoscape software. This tool allows the extraction of non-redundant biological GO features, the fusion of those terms which share similar associated genes and their grouping according to their kappa score. GO analysis was performed with the following parameters: a GO tree interval between 3 and 8 levels; an inclusion criterion of at least 3 proteins and 4% of proteins per GO term, and a kappa score of 0.4 was selected; significance (*p* value) of protein entries association with each term was calculated using a two-side hypergeometric test and corrected with a Bonferroni step down. GO terms with a *p* value < 0.01 were included for the analysis. GO term grouping was carried out with 1 initial group size and a 50% genes group merge.

### ELISA assay

S100A7/psoriasin levels were determined by CircuLex S100A7/psoriasin ELISA kit (MBL International Corporation), following manufacturer’s instructions. Thirty-five additional pSS patients (AECG 2002), properly stratified in the three above defined groups were included in order to validate the differential expression of S100A7/psoriasin in whole saliva. Data were compared using the Mann–Whitney U test and a *p* value lower than 0.05 was considered as significant.

### Statistical analysis

The experimental data were analysed for statistical significance using R software (version 3.5.1) A Shapiro–Wilk test was used to assess the normality of data distribution. Level of significance between pSS groups was addressed by performing a Student’s two-tailed t-test. *p* values were corrected using the Benjamini–Hochberg procedure as post hoc test, in order to minimize any type I errors and thus the occurrence of false positives. Proteins were considered significant and differentially expressed with a *p* value lower than 0.05 and a fold change higher than 2 (− 2 > FC >+ 2).

## Results

### Patient features

**Fig. 1:**
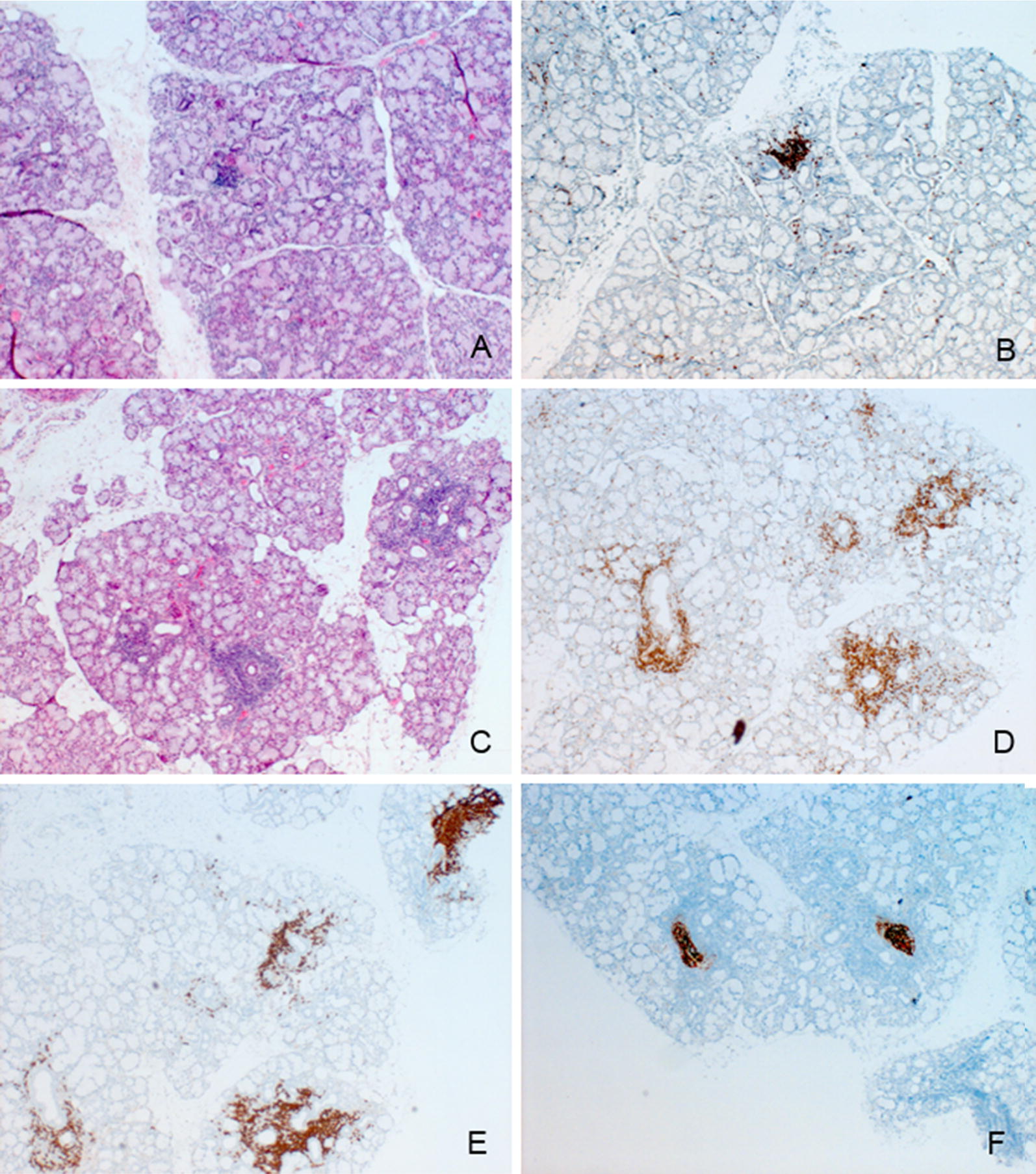
Immunohistochemical characterization of different subsets of pSS phenotypes. Representative images of different subsets of pSS: MSGB characterized by a low focus score (**A** *H&E* stain, magnification ×4) with T-*cell* marker (*CD3*) *immunohistochemically* evaluated (**B**) and MSGB characterized by a high focus score (**C**, *H&E* stain, magnification ×4) with *the inflammatory cell infiltrate evaluation* [*CD3* (**D**)*, CD20* (**E**) *and CD21* (**F**)]

Twenty pSS patients and 20 age- and sex-matched healthy volunteers were included in the study. As defined in Methods, we subgrouped three pSS patients phenotypes who differed for sialometry and MSGB focus score. Patients’ demographic and clinical features are summarized in Additional file 1: Table S1. Patients presenting high focus/normal flow were significantly younger when compared to those included in the other groups. No further differences were detected between the three groups. Figure 1 shows representative images of MSGBs characterized by different focus score and infiltrate composition severity. Figure 1A, B show respectively the H&E stain of a MSGB with lower focus score and the relative lymphocytic infiltration, mainly consisting of T-cells. Figure 1C–F depicts a more severe lesion with higher focus score and a more complex infiltration characterized by T-cells (1D), B-cells (1E) and germinal centers (1F).

### Saliva proteomics profiling

Comparison between the three pSS phenotypes and healthy controls was carried out by using the normalized protein abundances derived from the integration of all the peptide-specific extract ion chromatograms fragment ions for each matched protein.

A total of 674 proteins were identified in the ion spectral library with a FDR lower than 1%. Among these detected proteins, 302 proteins were quantified across all samples with a Protein Confidence higher than 95% and with a local false discovery rate lower than 1%, as stringent criterion to avoid false positives (Additional file 2: Table S2).

**Fig. 2:**
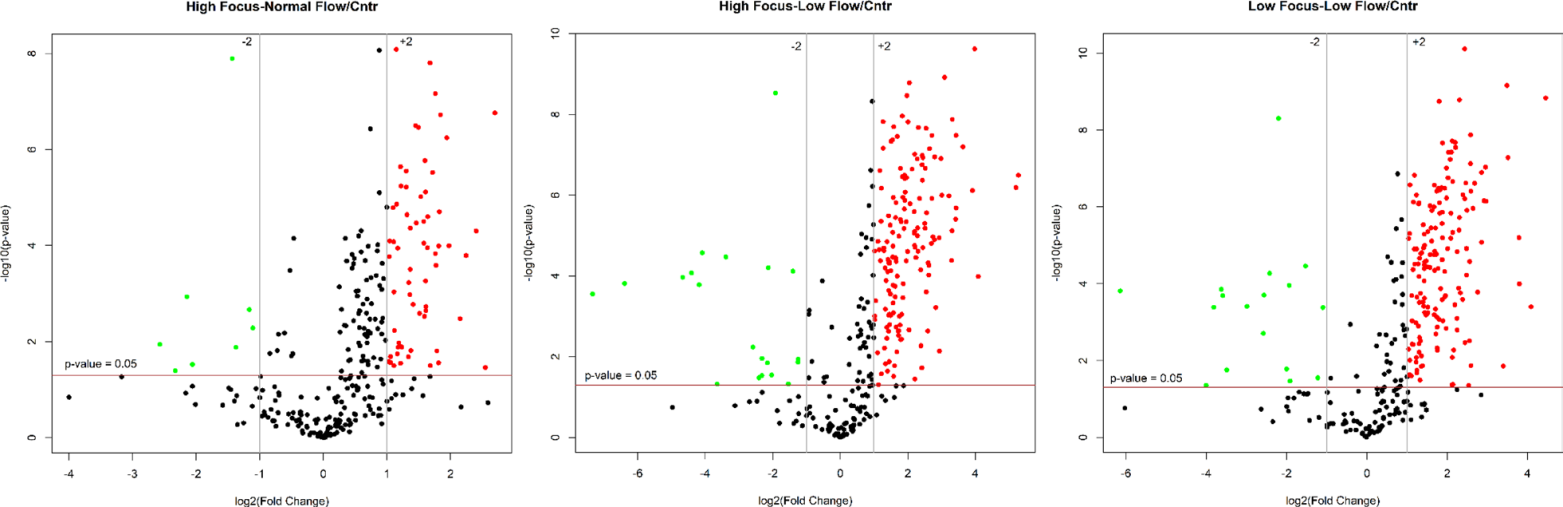
Comparison between pSS phenotypes and controls. Volcano plots show the differentially expressed proteins up- and down-regulated (fold change threshold = 2) among pSS phenotypes High Focus/Normal Flow, High Focus/Low Flow and Low Focus/Low Flow relative to controls

203 proteins resulted differently expressed in pSS patients with respect to control group. The majority of those proteins were up-regulated in pSS patients compared to controls. Particularly, 21.5%, 51.3% and 53.6% of proteins were found to be over-expressed (FC >+ 2, *p* < 0.05) in pSSpatients with high focus/normal flow, high focus/low flow and low focus/low flowrespectively, compared to controls. On the other hand, the level of down-regulated (FC < − 2, *p* < 0.05) proteins was significantly low with 2.6%, 6.6% and 5.6% for pSS patients with high focus/normal flow, high focus/low flow and low focus/low flow, respectively (Fig. 2).

**Fig. 3:**
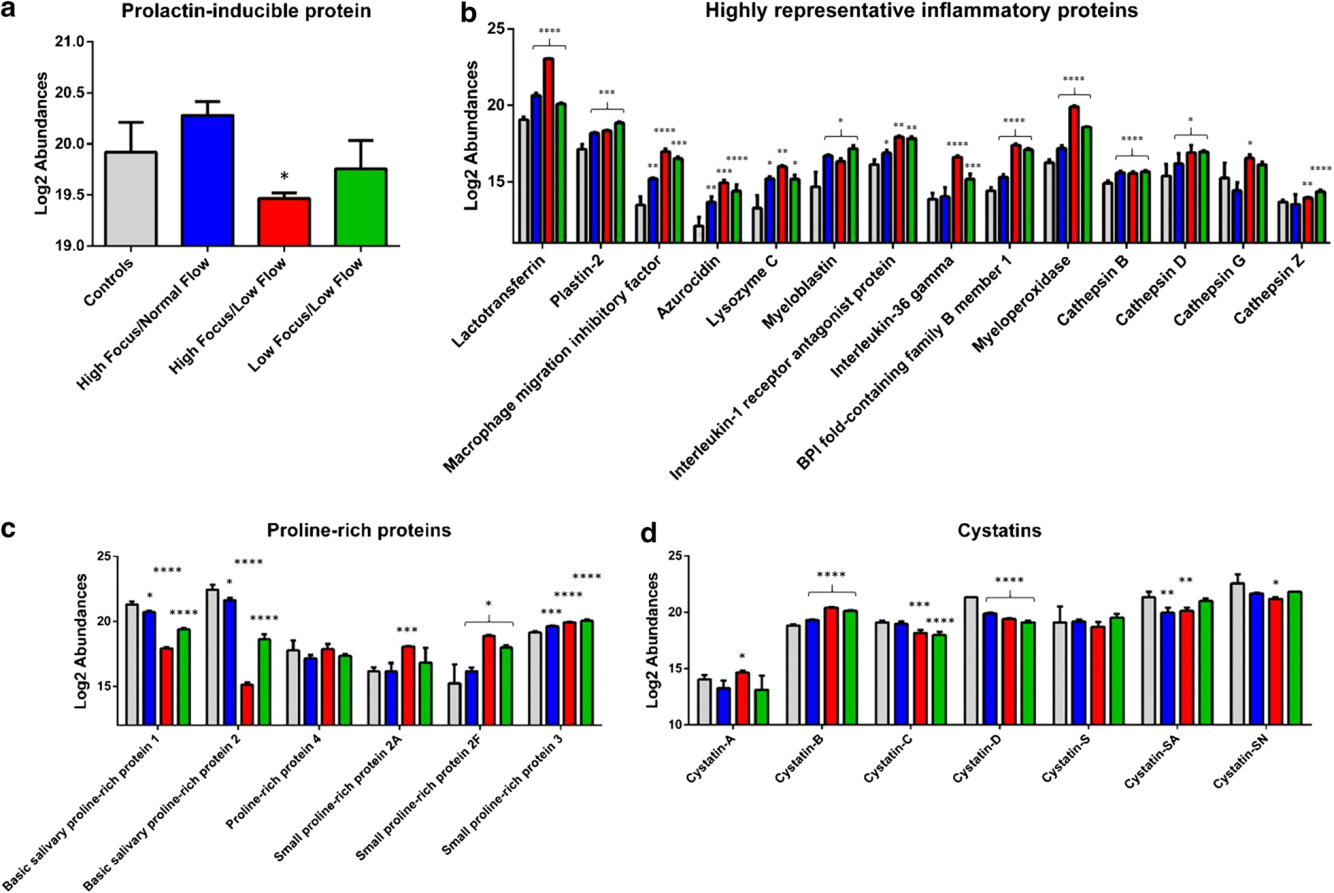
Significant differentially expressed proteins. Bar plots show the relative protein abundances between controls (grey) and High Focus/Normal Flow (blue), High Focus/Low Flow (red) and Low Focus/Low Flow (green) pSS groups of prolactine-inducible protein (**a**), proteins involved in the inflammatory process (**b**); protein family members of proline-rich proteins (**c**) and Cystatins (**d**). Difference significance was expressed as follows: *p < 0.05; **p < 0.01; ***p < 0.005, ****p < 0.001

We found evident differences between control group and the three pSS phenotypes regarding the expression of proteins that are normal components of the human salivary proteome, including: prolactin-inducible protein (PIP), proline-rich proteins (PRPs), cystatins and several mediators of inflammatory processes, as well. In particular, PIP protein showed lower level of expression compared to control especially in pSS patients with high focus/low flow, while no significant variations were observed between patients with high focus/normal flow and low focus/low flow relative to controls (Fig. 3a). Some members of PRPs family such as, small proline-rich protein 3, 2A and 2F, together with proline-rich protein 4 showed a higher level of up-regulation in patients with high focus/low flow than patients with high focus/normal flow and low focus/low flow compared to controls, with the only exception for basic salivary proline-rich protein 1 and 2, which showed a reverse trend (higher in patients with high focus/normal flow and low focus/low flow compared to patients with high focus/low flow) (Fig. 3c). Cystatins were generally downregulated in patients with high focus/low flow compared to patients with high focus/normal flow and to some extent to patients with low focus/low flow with an exception for cystatin B and A that are expressed by oral mucosa and not by salivary glands and that showed a higher expression in patients with high focus/low flow than in the other patient phenotypes compared to control group (Fig. 3d). Interestingly, a large number of proteins differentially expressed among the three phenotypes of pSS were shown to be involved in the regulation of inflammatory processes (Fig. 3b). The majority of them showed a similar pattern of expression characterized by a significant increase in patients with high focus/low flow compared to the other pSS phenotypes, relative to controls. In particular, we observed that the levels of both lactotransferrin and macrophage migration inhibitory factor, which are implicated in the activation of NF-kB pathway through LPS signaling, were drastically higher in patients with high focus/low flow. To the same extent, two members of the interleukin family, namely interleukin 1 receptor agonist and interleukin 36 gamma, and plastin-2 which altogether participate to the interleukin 12 mediated signaling pathway, were found to be up-regulated in patients with high focus/low flow and in those with low focus/low flow, respectively. Moreover, proteins involved in the antimicrobial humoral response such as Myeloperoxidase, Azurocidin, Lysozyme and Bactericidal fold-containing family B member 1, showed all the same expression trend with higher levels in patients with high focus/low flow. Finally, different members of cathepsin family involved in the regulation of complement activation, were found to be up-regulated in all the three phenotype groups (Cathepsin B and D) compared to control group, while Cathepsin G and Z showed a significant increase in patients with high focus/low flow and in those with low focus/low flow, respectively, compared to control group.

### Comparative analysis of different pSS phenotypes: do S100 proteins have a role in disease profiling?

**Fig. 4:**
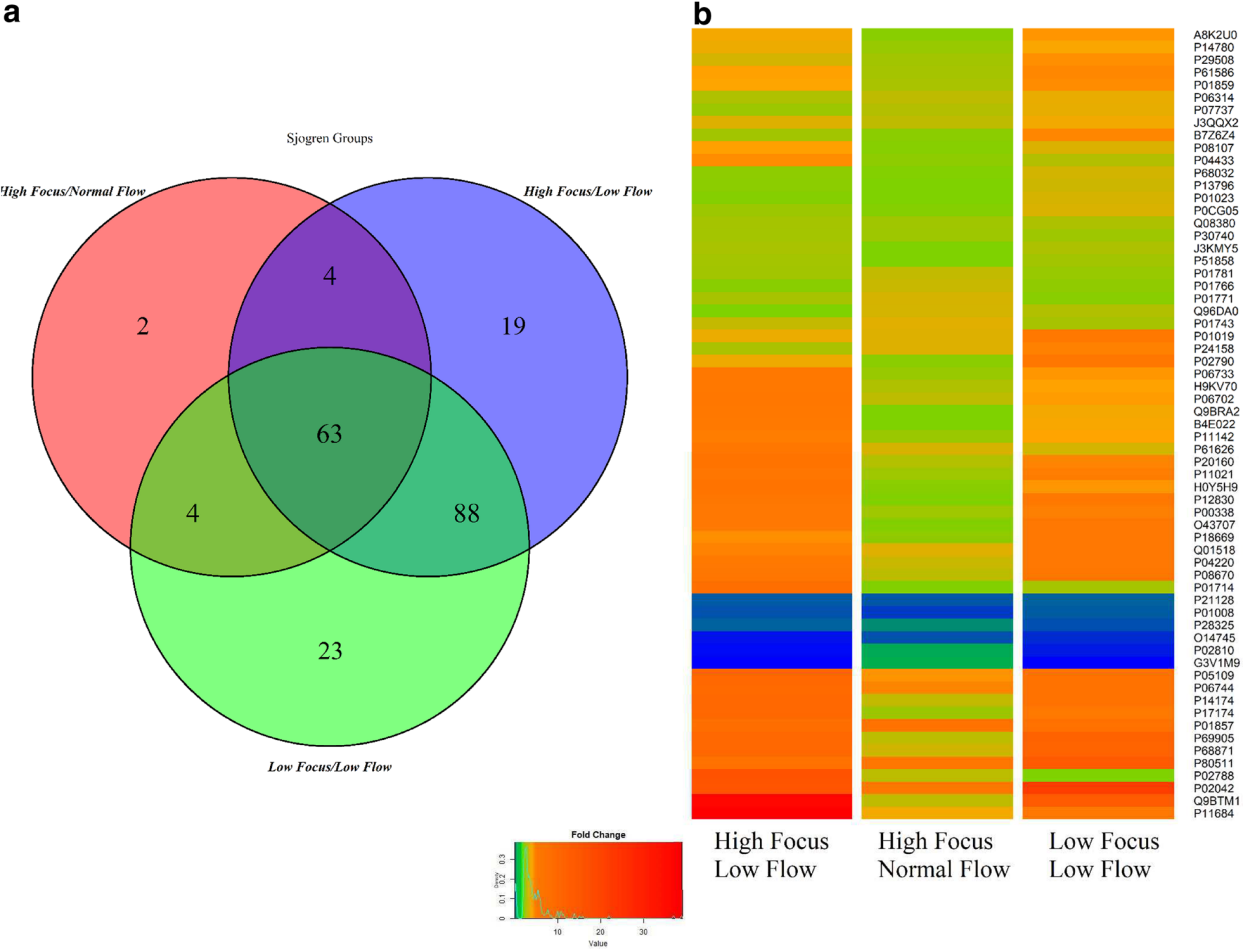
Differences among pSS phenotypes. Venn diagram of the differentially expressed proteins detected among phenotypes High Focus/Normal Flow, High Focus/Low Flow and Low Focus/Low Flow of pSS disease (**a**). Heat map showing the differences in terms of protein expression of the 63 common proteins among pSS phenotypes. High Focus/Low Flow and Low Focus/Low Flow patient groups showed the highest level of up-regulated proteins compared to High Focus/Normal Flow group (**b**)

Among the overall differentially expressed proteins, the three pSSgroups shared 63 proteins, whereas 19 and 23 were uniquely found in in patients with high focus/low flow and in those with low focus/low flow, respectively (Fig. 4a, Additional file 3: Table S3). The level of variation of the 63 proteins commonly identified in all three pSS phenotypes were compared in order to have an overall view of the main differences between the three subsets. As evidenced by the heat map, patients with high focus/low flow and in those with low focus/low flow, showed a higher level of up-regulation compared to patients with high focus/normal flow, indicating a clear variability among different phenotypes of the same disease (Fig. 4b).

**Fig. 5:**
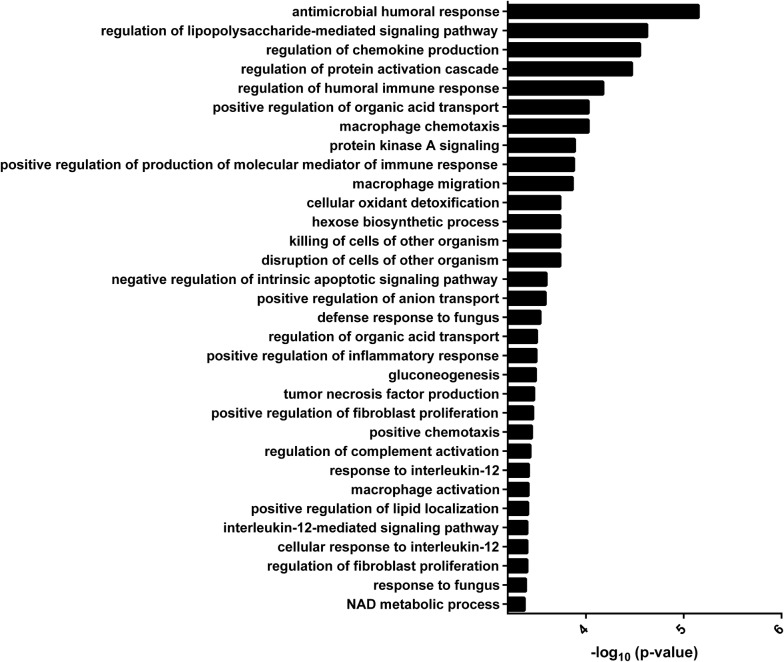
Gene ontology (GO) analysis for biological processes. A significant enrichment of canonical pathways related to inflammatory response was evidenced for the 63 differentially expressed proteins found to be in common between the three pSS phenotypes described in this study

**Fig. 6:**
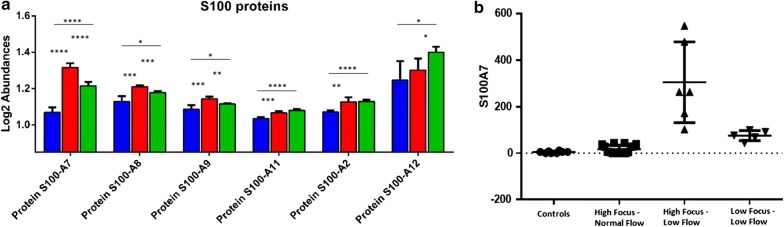
Proteomic profiling of S100 proteins. Different response of S100 family members was evidenced across High Focus/Normal Flow (blue), High Focus/Low Flow (Fleissig et al.) and Low Focus/Low Flow (green) pSS groups. (**a**); ELISA assay validated the MS data. Figure shows the concentration (expressed in ng/ml) of S 100 A7 in controls and the three pSS phenotypes (**b**); *p < 0.05; **p < 0.01; ***p < 0.005; ****p < 0.001

In order to understand the functional network in which these proteins belong to, GO enrichment analysis was carried out for biological processes (Fig. 5). Results showed a significant enrichment of GO terms associated to the regulation of immune response, chemokine production and secretion, chemotaxis, macrophage activation and in general to several other pathways that regulate the inflammatory processes. Accordingly, the family of S-100 proteins were found to co-localize in almost all the canonical pathway associated to the regulation of inflammatory response, including IL-12 signaling, thus indicating a key role of this class of proteins in this biological process. Of note, the level of S-100 A2, A7, A8 and A9 was shown to be higher in in patients with high focus/low flow, while S-100 A11 and A12 appeared significantly increased in patients with low focus/low flow, (Fig. 6a). Due to the potential pathogenetic role of S100A proteins in pSS and in the production of other inflammatory mediators, including IL-1 family proteins, we validated the expression of S100 A7 in 19 additional pSS patients and 8 controls confirming that S100A7 expression was significantly higher in patients with high focus/low flow (305.6 ± 174 ng/ml vs 11 ± 14 ng/ml vs 75.7 ± 21.6 ng/ml, *p* = 0.000) (Fig. 6b).

## Discussion

In this proof of concept study we identified several candidate salivary biomarkers able to distinguish patients with pSS from healthy controls and also to characterize different subsets of the disease. A number of specific protein families resulted as differently expressed in the three subgroups stratified according to patients unstimulated salivary flow rate and MSGB focus score. We speculated that these proteins may represent potential candidate biomarkers for disease specific pathogenic pathways and pSS clinical subsets.

In patients with established pSS and decreased salivary flow (i.e. both patients with high focus/low flow and low focus/low flow), proteomics analysis showed a considerable reduction of the normal constituent of saliva (i.e. PRPs, cystatins, GCDFP15/PIP, carboanhydrase VI) directly correlated to the impairment of salivary flow and to the salivary gland damage. In particular, patients with high focus/low flow expressed at the highest level inflammation proteins, thus mirroring salivary gland infiltration. Moreover, another class of proteins, specifically increased in these patients with low salivary flow, was represented by inflammatory proteins including MPO, H4 and cathepsin G. These proteins exert a pivotal role in the modulation of oral microbiota but their functional role remained enigmatic. In this study we enrolled patients without an overt periodontitis, however, we used whole saliva therefore we cannot exclude that these proteins may reflect the alteration of oral environment.

As expected, considering the preserved salivary flow and the significant younger age at the diagnosis, patients with high focus/normal flow presented a less remarked reduction of the normal constituent of saliva. A likely hypothesis is that those patients are characterized by an early pSS phenotype that may be favored towards the evolution into the other subgroups. From this perspective the observed alterations may be suggested as early biomarker of the disease. However, since we used a cross-sectional study design in the absence of longitudinal prospective data, it remains debatable whether the three subsets may evolve one into the other over the time.

A point of strength of the study was the adopted work-flow strategy. Proteomic studies carried out in pSS in the past few years, have focused on highly abundant proteins, normal components of whole saliva. In the current work we applied a immunodepletion protocol to remove albumin and IgGin order to unmask potential low-abundance biomarkers. A similar immunodepletion protocol was used in 2015 by Deutsch et al. [22]. These authors by applying 2D-PAGE and quantitative dimethylation liquid chromatography tandem mass spectrometry (LC-MS/MS) described 61 proteins differentially expressed in pSS vs healthy controls. In our work, instead of 2D-PAGE we exploited a nano-HPLC system coupled with a TripleTOF 5600+ mass spectrometer. This high throughput technique allowed the identification of 133 differentially expressed proteins. Thus, in addition to normal components of saliva, we were able to characterize proteins involved in oral innate and adaptive immune response [31].

Interestingly GO analysis showed a significant enrichment of canonical pathways related to antimicrobial and inflammatory response in all of the three pSS subsets thus indicating that potential biomarker of disease could be fished among them.

We specifically focused on S100A proteins that have been reported to be increased in saliva and serum of pSS [32] and associated also to a high risk of vascular complications in pSS patients [33–35]. S100 A proteins converged on IL-12 signaling at the GO analysis, a biological process that has been demonstrated to play active roles in the expansion and organization of infiltrative injuries in SS, mirroring specifically DC involvement and Th1 cells differentiation in the disease pathogenesis [6, 36]. We found that S100A proteins were expressed abundantly in saliva, at local sites of inflammation, and we also observed their increase in a stage-manner from pSS-phenotype with normal flow to pSS-phenotype with low flow. These observations lead us to speculate that these proteins may be key factors in the progression of pSS glandular dysfunction and oral involvement and can be used as biomarkers for pSS.

Infact, among S100 A proteins, S100A7, S100A8, S100A9, S100A12 have been already described as differentially expressed in other systemic autoimmune diseases and particularly in Systemic Sclerosis [11, 37–40]. Moreover, increased S100A7 has also been observed in oral dysplasia thus arising the possibility that the oral epithelium might be the source of expression for them, also in pSS [41].

However, although the exact role of S100 proteins in the pathogenesis of pSS is notyet clear, their increased expression in infiltrating cells and around blood vessel walls has been demonstrated, thus suggesting that they might be functionally relevant in the pathogenesis ofthe disease [42–44]. Indeed, S100 proteins are able to interact with TLR4 and to induce a TLR4-dependent NF-κB activation and a pro-inflammatory cytokine response in monocytes [45, 46].

We acknowledge that our results need to be validated in a larger population including not only pSS patients but also different disease control groups (i.e. patients with HCV infection or sarcoidosis). Moreover, further studies are necessary to deeply investigate the role of S100 proteins in pSS pathogenesis. However, this proof of concept study reinforces the role of salivary proteomics in profiling different subsets of pSS and the potentiality of this approach in unveiling pathophysiologic processes underlying glandular inflammation and dysfunction.

## Conclusions

In this study we have explored the potentialities of salivary proteomics to identify pSS different phenotypes. The originality of our work stands in the use of a powerful LC-SWATH-MS technology to the analysis of a complex and heterogeneous systemic disease such as pSS. This approach allowed the identification of a panel of differently expressed proteins that seems apparently able to distinguish specific subsets of pSS patients. Some of the identified proteins apparently reflect the inflammation and damage that characterize pSS. Other proteins such as S100 proteins have a more defined role in the pathogenesis of the disease. Therefore, these findings, once evaluated in larger studies, may represent the first step towards the identification of reliable biomarkers, useful to stratify homogeneous subsets of patients. The identification of S100 proteins as potential pSS early biomarkers for pSS glandular inflammation and dysfunction may improve the assessment of the disease, ultimately fostering the development of novel tailored treatments.

## Additional files

**Additional file 1: Table S1.** Patients demographic and clinical features grouped by inflammation grade and salivary flow rate

**Additional file 2: Table S2.** Quantitative data from the three different stages of Sjogren disease (High Focus/Normal Flow, High Focus/Low Flow and Low Focus/Low Flow). Protein abundances are expressed as average between biological and technical replicates. Fold changes were calculated relatively to control group. Reproducibilty was expressed as %CV.

**Additional file 3: Table S3.** Differentially expressed proteins found in common between High Focus/Normal Flow, High Focus/ Low Flow and Low Focus/Low Flow groups. Fold changes were showed per each protein.

## Data Availability

All data generated or analysed during this study are included in this published article and its supplementary information files.

## References

[1] Tzioufas AG, Vlachoyiannopoulos PG (2012). Sjogren’s syndrome: an update on clinical, basic and diagnostic therapeutic aspects. J Autoimmun.

[2] Brito-Zeron P, Baldini C, Bootsma H, Bowman SJ, Jonsson R, Mariette X (2016). Sjogren syndrome. Nat Rev Dis Primers.

[3] Baldini C, Ferro F, Luciano N, Bombardieri S, Grossi E (2018). Artificial neural networks help to identify disease subsets and to predict lymphoma in primary Sjögren’s syndrome. Clin Exp Rheumatol.

[4] Argyropoulou OD, Valentini E, Ferro F, Leone MC, Cafaro G, Bartoloni E (2018). One year in review 2018: Sjögren’s syndrome. Clin Exp Rheumatol.

[5] Goules AV, Tzioufas AG (2019). Lymphomagenesis in Sjögren’s syndrome: predictive biomarkers towards precision medicine. Autoimmun Rev.

[6] Manoussakis MN, Boiu S, Korkolopoulou P, Kapsogeorgou EK, Kavantzas N, Ziakas P (2007). Rates of infiltration by macrophages and dendritic cells and expression of interleukin-18 and interleukin-12 in the chronic inflammatory lesions of Sjogren’s syndrome: correlation with certain features of immune hyperactivity and factors associated with high risk of lymphoma development. Arthritis Rheum.

[7] Christodoulou MI, Kapsogeorgou EK, Moutsopoulos HM (2010). Characteristics of the minor salivary gland infiltrates in Sjogren’s syndrome. J Autoimmun.

[8] Kroese FGM, Haacke EA, Bombardieri M (2018). The role of salivary gland histopathology in primary Sjögren’s syndrome: promises and pitfalls. Clin Exp Rheumatol.

[9] Baldini C, Gallo A, Perez P, Mosca M, Alevizos I, Bombardieri S (2012). Saliva as an ideal milieu for emerging diagnostic approaches in primary Sjogren’s syndrome. Clin Exp Rheumatol.

[10] Gallo A, Baldini C, Teos L, Mosca M, Bombardieri S, Alevizos I (2012). Emerging trends in Sjogren’s syndrome: basic and translational research. Clin Exp Rheumatol.

[11] Baldini C, Cecchettini A, Gallo A, Bombardieri S (2017). Updates on Sjogren’s syndrome: from proteomics to protein biomarkers. Expert Rev Proteomics.

[12] Baldini C, Ferro F, Elefante E, Bombardieri S (2018). Biomarkers for Sjögren’s syndrome. Biomark Med.

[13] Ryu OH, Atkinson JC, Hoehn GT, Illei GG, Hart TC (2006). Identification of parotid salivary biomarkers in Sjogren’s syndrome by surface-enhanced laser desorption/ionization time-of-flight mass spectrometry and two-dimensional difference gel electrophoresis. Rheumatology.

[14] Giusti L, Baldini C, Bazzichi L, Bombardieri S, Lucacchini A (2007). Proteomic diagnosis of Sjogren’s syndrome. Expert Rev Proteomics.

[15] Giusti L, Baldini C, Bazzichi L, Ciregia F, Tonazzini I, Mascia G (2007). Proteome analysis of whole saliva: a new tool for rheumatic diseases–the example of Sjogren’s syndrome. Proteomics.

[16] Hu S, Wang J, Meijer J, Ieong S, Xie Y, Yu T (2007). Salivary proteomic and genomic biomarkers for primary Sjogren’s syndrome. Arthritis Rheum.

[17] Fleissig Y, Deutsch O, Reichenberg E, Redlich M, Zaks B, Palmon A (2009). Different proteomic protein patterns in saliva of Sjogren’s syndrome patients. Oral Dis.

[18] Baldini C, Giusti L, Ciregia F, Da Valle Y, Giacomelli C, Donadio E (2011). Proteomic analysis of saliva: a unique tool to distinguish primary Sjogren’s syndrome from secondary Sjogren’s syndrome and other sicca syndromes. Arthritis Res Therapy.

[19] Baldini C, Giusti L, Ciregia F, Da Valle Y, Giacomelli C, Donadio E (2011). Correspondence between salivary proteomic pattern and clinical course in primary Sjogren syndrome and non-Hodgkin’s lymphoma: a case report. J Transl Med.

[20] Ambatipudi KS, Swatkoski S, Moresco JJ, Tu PG, Coca A, Anolik JH (2012). Quantitative proteomics of parotid saliva in primary Sjogren’s syndrome. Proteomics.

[21] Tzioufas AG, Kapsogeorgou EK (2015). Biomarkers. Saliva proteomics is a promising tool to study Sjogren syndrome. Nat Rev Rheumatol.

[22] Deutsch O, Krief G, Konttinen YT, Zaks B, Wong DT, Aframian DJ (2015). Identification of Sjogren’s syndrome oral fluid biomarker candidates following high-abundance protein depletion. Rheumatology.

[23] Delaleu N, Mydel P, Kwee I, Brun JG, Jonsson MV, Jonsson R (2015). High fidelity between saliva proteomics and the biologic state of salivary glands defines biomarker signatures for primary Sjogren’s syndrome. Arthritis Rheumatol.

[24] Delaleu N, Mydel P, Brun JG, Jonsson MV, Alimonti A, Jonsson R (2016). Sjogren’s syndrome patients with ectopic germinal centers present with a distinct salivary proteome. Rheumatology.

[25] Martini D, Gallo A, Vella S, Sernissi F, Cecchettini A, Luciano N (2017). Cystatin S-a candidate biomarker for severity of submandibular gland involvement in Sjögren’s syndrome. Rheumatology (Oxford).

[26] Goules AV, Tzioufas AG (2016). Primary Sjogren’s syndrome: clinical phenotypes, outcome and the development of biomarkers. Immunol Res.

[27] Vitali C, Bombardieri S, Jonsson R, Moutsopoulos HM, Alexander EL, Carsons SE (2002). Classification criteria for Sjogren’s syndrome: a revised version of the European criteria proposed by the American-European Consensus Group. Ann Rheum Dis.

[28] Fisher BA, Jonsson R, Daniels T, Bombardieri M, Brown RM, Morgan P (2016). Standardisation of labial salivary gland histopathology in clinical trials in primary Sjogren’s syndrome. Ann Rheumatic Dise.

[29] Risselada AP, Kruize AA, Goldschmeding R, Lafeber FP, Bijlsma JW, van Roon JA (2014). The prognostic value of routinely performed minor salivary gland assessments in primary Sjogren’s syndrome. Ann Rheum Dis.

[30] Bindea G, Mlecnik B, Hackl H, Charoentong P, Tosolini M, Kirilovsky A (2009). ClueGO: a Cytoscape plug-into decipher functionally grouped gene ontology and pathway annotation networks. Bioinformatics.

[31] Delgado-Rizo V, Martinez-Guzman MA, Iniguez-Gutierrez L, Garcia-Orozco A, Alvarado-Navarro A, Fafutis-Morris M (2017). Neutrophil extracellular traps and its implications in inflammation: an overview. Front Immunol.

[32] Nordal HH, Brun JG, Hordvik M, Eidsheim M, Jonsson R, Halse AK (2016). Calprotectin (S100A8/A9) and S100A12 are associated with measures of disease activity in a longitudinal study of patients with rheumatoid arthritis treated with infliximab. Scand J Rheumatol.

[33] Balarini GM, Zandonade E, Tanure L, Ferreira GA, Sardenberg WM, Serrano EV (2016). Serum calprotectin is a biomarker of carotid atherosclerosis in patients with primary Sjogren’s syndrome. Clin Exp Rheumat.

[34] Brun JG, Cuida M, Jacobsen H, Kloster R, Johannesen AC, Hoyeraal HM (1994). Sjogren’s syndrome in inflammatory rheumatic diseases: analysis of the leukocyte protein calprotectin in plasma and saliva. Scand J Rheumatol.

[35] Nordal HH, Brun JG, Halse AK, Madland TM, Fagerhol MK, Jonsson R (2014). Calprotectin (S100A8/A9), S100A12, and EDTA-resistant S100A12 complexes (ERAC) in primary Sjogren’s syndrome. Scand J Rheumatol.

[36] Liaskou E, Patel SR, Webb G, Bagkou Dimakou D, Akiror S, Krishna M (2018). Increased sensitivity of Treg cells from patients with PBC to low dose IL-12 drives their differentiation into IFN-γ secreting cells. J Autoimmun.

[37] Anjana R, Joseph L, Suresh R (2012). Immunohistochemical localization of CD1a and S100 in gingival tissues of healthy and chronic periodontitis subjects. Oral Dis.

[38] Kojima T, Andersen E, Sanchez JC, Wilkins MR, Hochstrasser DF, Pralong WF (2000). Human gingival crevicular fluid contains MRP8 (S100A8) and MRP14 (S100A9), two calcium-binding proteins of the S100 family. J Dent Res.

[39] Austermann J, Spiekermann C, Roth J (2018). S100 proteins in rheumatic diseases. Nat Rev Rheumatol.

[40] Baldini C, Giusti L, Bazzichi L, Ciregia F, Giannaccini G, Giacomelli C (2008). Association of psoriasin (S100A7) with clinical manifestations of systemic sclerosis: is its presence in whole saliva a potential predictor of pulmonary involvement?. J Rheumatol.

[41] Hwang JT, Gu YR, Shen M, Ralhan R, Walfish PG, Pritzker KP (2017). Individualized five-year risk assessment for oral premalignant lesion progression to cancer. Oral Surg Oral Med Oral Pathol Oral Radiol.

[42] Nicaise C, Weichselbaum L, Schandene L, Gangji V, Dehavay F, Bouchat J (2017). Phagocyte-specific S100A8/A9 is upregulated in primary Sjogren’s syndrome and triggers the secretion of pro-inflammatory cytokines in vitro. Clin Exp Rheumatol.

[43] Cuida M, Brun JG, Johannessen AC, Jonsson R (1996). Immunohistochemical characterization of the cellular infiltrates in Sjogren’s syndrome, rheumatoid arthritis and osteoarthritis with special reference to calprotectin-producing cells. APMIS: Acta Pathologica, Microbiologica, et Immunologica Scandinavica.

[44] Cuida M, Brun JG, Tynning T, Jonsson R (1995). Calprotectin levels in oral fluids: the importance of collection site. Eur J Oral Sci.

[45] Chen B, Miller AL, Rebelatto M, Brewah Y, Rowe DC, Clarke L (2015). S100A9 induced inflammatory responses are mediated by distinct damage associated molecular patterns (DAMP) receptors in vitro and in vivo. PLoS ONE.

[46] He Z, Riva M, Bjork P, Sward K, Morgelin M, Leanderson T (2016). CD14 is a co-receptor for TLR4 in the S100A9-induced pro-inflammatory response in monocytes. PLoS ONE.

