# Supplementary material for: Phenotyping multiple subsets in Sjögren’s syndrome: a salivary proteomic SWATH-MS approach towards precision medicine
Source: Clin Proteomics. 2019 Jun 20;16:26. doi: 10.1186/s12014-019-9245-1 (PMC6587286; doi:10.1186/s12014-019-9245-1)
Supplement: Supplementary file 1 — Additional file 1: Table S1. Patients demographic and clinical features grouped by inflammation grade and salivary flow rate [file 12014_2019_9245_MOESM1_ESM.docx]

**Additional file 1: Table S1. Patients demographic and clinical features grouped by inflammation grade and salivary flow rate**

|  | **pSS-High Focus/Normal Flow (n=8)** | **pSS-High Focus/Low Flow (n=7)** | **pSS-Low Focus/Low Flow (n=5)** | **p-value** |
| --- | --- | --- | --- | --- |
| **Age (mean(SD))** | 41 (13) | 66 (8) | 55(13) | 0.002 |
| **Disease duration (mean(SD))** | 3.6 (3.9) | 3.8(1.4) | 2.6 (2.2) | ns |
| **Focus score (mean(SD))** | 3.4 (1.6) | 3.1 (0.8) | 1.4(1.1) | 0.03 |
| **USFR (mean(SD))** | 4.2 (1.2) | 0.7 (0.4) | 0.9 (0.7) | 0.000 |
| **Xerostomia** | 6/8 (75%) | 6/7 (85.7%) | 5/5 (100%) | n.s |
| **Xerophtalmia** | 8/8 (100%) | 6/7 (85.7%) | 5/5 (100%) | n.s |
| **Ocular Tests** | 7/8 (87.5%) | 6/7 (85.7%) | 5/5 (100%) | n.s |
| **ANA** | 8/8 (100%) | 7/7 (100%) | 5/5 (100%) | n.s |
| **Anti-Ro/SSA** | 7/8 (87.5%) | 6/7 (85.7%) | 2/5 (40%) | n.s |
| **Anti-La/SSB** | 4/8 (50%) | 3/7 (42.9%) | 1/5 (20%) | n.s |
| **RF** | 4/8 (50%) | 2/7 (28.6%) | none | n.s |
| **Cryo** | 1/8 (12.5%) | none | none | n.s |
| **GC-like structures** | 4/8 (50%) | 4/7 (57.1%) | none | n.s |
| **ESSDAI (mean(SD))** | 7(5) | 5(3) | 0(0) | 0.04 |

Notes: USFR: Unstimulated, salivary, flow rate; n.s = not significant; ANA: antinuclear antibodies; RF: rheumatoid Factor; Cryo: cryoglobulins; GC-like structures: germinal-center-like structures; ESSDAI: Eular Sjögren’s syndrome disease activity index
